# Evaluation of Oncology Trial Results Reporting Over a 10-Year Period

**DOI:** 10.1001/jamanetworkopen.2021.10438

**Published:** 2021-05-24

**Authors:** Xu Liu, Yuan Zhang, Wen-Fei Li, Everett Vokes, Ying Sun, Quynh-Thu Le, Jun Ma

**Affiliations:** 1 Department of Radiation Oncology, Sun Yat-sen University Cancer Center, State Key Laboratory of Oncology in South China, Collaborative Innovation Center of Cancer Medicine, Guangzhou, Guangdong, China; 2 Department of Medicine, University of Chicago Medicine and Biological Sciences, Chicago, Illinois; 3 Department of Radiation Oncology, Stanford University, Stanford, California

## Abstract

**Importance:**

Unreported clinical trial results represent a violation of human rights. Oncology trials account for nearly 30% of interventional biopharmaceutical clinical studies registered on ClinicalTrials.gov and are the most numerous among all disciplines.

**Objective:**

To analyze the reporting of results among all interventional oncology trials registered on ClinicalTrials.gov from 2007 through 2017.

**Design, Setting, and Participants:**

This cohort study analyzed all clinical studies registered between June 1, 2007, and May 8, 2017, on ClinicalTrials.gov, the largest public clinical trial registry in the world. Trials with a recruitment status of completed or terminated and a primary completion date of on or before September 30, 2017, were selected. Data were analyzed between February 20, 2021, and February 26, 2021.

**Main Outcomes and Measures:**

The main outcome was the percentage of trials that reported results either on ClinicalTrials.gov or in journal publications within 24 months of the primary completion date. Journal publication was ascertained by searching ClinicalTrials.gov for a link to the publication, PubMed using national clinical trial number, and Embase using national clinical trial number and filters.

**Results:**

Of the 12 240 clinical trials registered in ClinicalTrials.gov, 7425 trials (60.7%; 95% CI, 60.0%-61.5%) reported results, with a 34.0% (95% CI, 30.3%-37.7%) increase in 24-month reporting rate from 2007 to 2017. Multivariable analyses confirmed that more recent trials (adjusted hazard ratio [HR], 1.11 per year increase; 95% CI, 1.10-1.13) and trials with larger sample sizes (51-100 patients: adjusted HR, 1.17 [95% CI, 1.09-1.24]; >100 patients: adjusted HR, 1.43 [95% CI, 1.33-1.54]) were more likely to report results. Terminated trials were less likely to report results compared with completed trials (adjusted HR, 0.88; 95% CI, 0.83-0.93). Compared with trials funded by industry, those funded by the National Institutes of Health were more likely to report results (adjusted HR, 1.39; 95% CI, 1.29-1.49), whereas those funded by other academic or nonprofit organizations were less likely to report results (adjusted HR, 0.66; 95% CI, 0.62-0.70). Among all 7425 trials, the results of 2807 trials (37.8%; 95% CI, 36.7%-38.9%) were posted only on ClinicalTrials.gov. These trials tended to be terminated early and to have small sample sizes (≤50 patients) compared with trials that published results in journals.

**Conclusions and Relevance:**

This study found a gradual improvement in results reporting among oncology trials over a 10-year period. Trial registries could serve as a results reporting platform for unpublished trials and as a data source of trial outcomes for future studies.

## Key Points

**Question:**

What percentage of interventional oncology trials registered on ClinicalTrials.gov from 2007 through 2017 have reported results?

**Findings:**

In this cohort study of 12 240 completed or terminated oncology trials registered with ClinicalTrials.gov, only 7425 (60.7%) reported results on ClinicalTrials.gov and/or in journal articles. The 24-month reporting rate for trials completed or terminated in or before 2007 was 5.1% and increased to 39.1% in 2017.

**Meaning:**

This study found an increase in the reporting of oncology trial results over 10 years and a basis for future improvement.

## Teaser

This cohort study examines the rate of results reporting among interventional cancer trials registered on ClinicalTrials.gov from 2007 to 2017.

## Introduction

Researchers who conduct clinical trials involving human experimentation have an ethical obligation to make their findings publicly available. However, numerous examples exist of potentially harmful data being withheld from the public and/or selective nonreporting.^1,2,3^ Possible reasons for nonpublication include lack of resources to prepare and submit manuscripts; limited journal space; and journal editors’ reluctance to publish certain trials, especially those that were terminated early and/or those with adverse outcomes.^4,5,6,7^ Because unreported trials represent a violation of human rights, in 2007, the Federal Drug Association Amendments Act (FDAAA), section 801, mandated that sponsors of trials meet specific criteria for reporting summary results on ClinicalTrials.gov, the largest and most robust trial registry in the world. This mandate has provided another source of clinical trial results data for widespread public access.

Cancer is the first or second leading cause of death in 91 countries,^8^ with oncology trials representing nearly 30% of interventional biopharmaceutical clinical studies registered on ClinicalTrials.gov.^6^ A 2013 study assessed the rate of results reporting among 646 oncology trials that were completed from 2007 through 2010.^9^ However, in the 2013 study, we included a limited number of trials and only trials that were subject to the policies of the FDAAA. The purpose of this study was to analyze the reporting of results among all interventional oncology trials registered on ClinicalTrials.gov from 2007 through 2017.

## Methods

### Study Selection and Database Annotation

This study is reported in accordance with the Guidelines for Reporting Meta-Epidemiological Methodology Research.^10^ We also followed the Strengthening the Reporting of Observational Studies in Epidemiology (STROBE) reporting guideline. The institutional review board of Sun Yat-sen University Cancer Center deemed this study exempt from review because it did not constitute human participant research and because no patient was involved.

As reported in a previous study, we downloaded all of the clinical studies registered on ClinicalTrials.gov as of May 8, 2017, using the Aggregate Analysis of ClinicalTrials.gov database.^11^ The database as well as data definitions and data dictionaries were available at the Clinical Trials Transformation Initiative website. We restricted our selection to interventional trials that were registered from June 1, 2007, to May 8, 2017, with a primary purpose of treatment and a focus on oncology. This selection process has been previously reported.^11^ Briefly, we selected trials with a recruitment status of completed or terminated and a primary completion date of on or before September 30, 2017. ClinicalTrials.gov defined the primary completion date as the date on which data collection was completed for all of the primary outcomes. This selection was intended to allow trial sponsors or investigators at least 2 years to report the results. The raw data have been uploaded to the Research Data Deposit.^12^

The database processing and annotation methods in this study have been reported previously.^11^ Funding sources were classified according to the lead sponsor and/or collaborator recorded for each clinical trial: National Institutes of Health (NIH), industry, or other academic or nonprofit organizations. If a company was listed as the lead sponsor or collaborator without the NIH, the trial was classified as an industry-funded trial. If the NIH was the lead sponsor or collaborator with a nonindustry lead sponsor, the trial was considered an NIH-funded trial. All other trials were considered as other-funded trials.^11,13,14^

### Study Outcomes, Status of Results Reporting, and Time to Reporting

The main outcome was the percentage of trials that reported results within 24 months after the primary completion date. We defined results reporting as results that were either published in journals or posted on ClinicalTrials.gov. To identify a trial’s publication status, we used a systematic 3-step search strategy. First, we ascertained whether a link to a publication was provided in the study’s records on ClinicalTrials.gov, which can be reported by the sponsor or investigator. ClinicalTrials.gov automatically searches MEDLINE daily and extracts all publications that have a matched ClinicalTrials.gov identifier or national clinical trial (NCT) number. Second, if no matching publications were returned, we queried the PubMed database using the NCT numbers, similar to the methods used in previous studies.^6,15^ We did not restrict the search field or use any filters in PubMed to maximize sensitivity. Third, we searched Embase using NCT numbers and filters ([article OR article in press OR letter] and [clinical study]).

We excluded abstracts from conference proceedings because the results provided in those types of abstracts have been found to be incomplete or premature.^16,17^ The articles identified were matched to the characteristics registered on ClinicalTrials.gov, including the condition studied, intervention, study design, primary investigators, locations, and primary outcomes. Two of us (Y.Z. and X.L.) separately conducted the search from October 1, 2019, to December 31, 2019, and updated our search in February 2021. We recorded the earliest publication date of a trial’s main results that reported at least 1 primary outcome. All questionable cases (partial matches) were resolved by consensus.

For all published trials, we used the time to publication as the time from the primary completion date to the date of publication. For publications available online ahead of print, we used the earlier online access date. For trials that posted results online on ClinicalTrials.gov, we established the time to post as the time from the primary completion date to the results first posted date. If a trial reported results both in a journal publication and on ClinicalTrials.gov, the time to reporting was calculated as the time from the primary completion date to the date of publication or the results first posted date, whichever occurred first. The follow-up time for trials not yet published was calculated as the time from the primary completion date to the start date of the updated search (February 1, 2021).

### Statistical Analysis

We assessed the percentage of trials that reported results overall and within 24 months of the primary completion date, which is consistent with previous studies.^7,18^ Descriptive statistics were used to summarize the characteristics of the clinical trials. Categorical variables were reported as counts and percentages. Unless stated otherwise, missing values were excluded from the analyses. Pearson χ^2^ test was used to compare trial characteristics, and Fisher exact test was used if indicated. For descriptive purposes, we used the Kaplan-Meier method to estimate the cumulative percentage of trials that reported results at monthly intervals from the primary completion date. We used a multivariable Cox proportional hazards regression model to examine the factors associated with the time to reporting. The model included 8 prespecified trial characteristics: primary completion year, registration before participant enrollment, phase, number of groups, treatment randomization, total enrollment, funding source, and recruitment status (terminated vs completed).

All analyses were performed using Stata, version 12.0 (StataCorp LLC). All tests were 2-tailed, and 2-sided *P* < .05 was considered to be statistically significant. Data were analyzed between February 20, 2021, and February 26, 2021.

## Results

### Trial Characteristics

Among the 243 758 clinical studies registered on ClinicalTrials.gov, 25 907 were interventional trials that were registered from June 1, 2007, to May 8, 2017, with treatment as the primary purpose and cancer as the focus. Of the 25 907 trials, 12 240 (47.2%) with a completed or terminated status and a primary completion date on or before September 30, 2017, were included in the analysis (eFigure 1 in the Supplement).

The characteristics of the 12 240 trials that were completed or terminated before October 1, 2017, are presented in Table 1, with a total of 1 138 004 participants enrolled in these trials. Approximately 2 of every 3 studies (7909 [64.6%]) were in phases 2 to 4, and roughly one-quarter (3327 [27.2%]) were in phases 0 to 1. The phases of the remaining trials (1004 [8.2%]) were categorized as not applicable, including trials of devices or behavioral interventions. More than half of all trials (6421 [52.5%]) were industry funded, and 1423 (11.6%) were NIH funded. Other academic or nonprofit organizations funded 4396 (35.9%) trials. A total of 9363 trials (76.5%) were completed, whereas 2977 trials (23.5%) were terminated prematurely.

**Table 1.** Characteristics of Included Trials

Characteristic	No. (%)^a^	Trials with any results reported, No. (%)
No.	12 240	NA
Registration before participant enrollment		
No	5282 (43.2)	2902 (54.9)
Yes	6951 (56.8)	4519 (65.0)
Phase		
0-1	3327 (27.2)	1416 (42.6)
1/2-2	6270 (51.2)	4352 (69.4)
2/3-3	1297 (10.6)	939 (72.4)
4	342 (2.8)	195 (57.0)
NA^b^	1004 (8.2)	523 (52.1)
No. of groups		
1	7140 (59.2)	4183 (58.6)
2	3699 (30.6)	2441 (66.0)
≥3	1231 (10.2)	749 (60.8)
Treatment randomization		
Nonrandomized	8337 (69.1)	4906 (58.8)
Randomized	3735 (30.9)	2450 (65.7)
Blinding		
Open label	10 854 (88.7)	6490 (59.8)
Any blinding	1386 (11.3)	935 (67.5)
Total enrollment, No. of patients		
≤50	7898 (64.6)	4395 (55.6)
51-100	2098 (17.2)	1360 (64.8)
>100	2224 (18.2)	1659 (74.6)
With US study site		
No	4499 (38.3)	2348 (52.2)
Yes	7242 (61.7)	4807 (66.4)
Funding source		
Industry	6421 (52.5)	4142 (64.5)
NIH	1423 (11.6)	982 (69.0)
Other academic or nonprofit organizations	4396 (35.9)	2301 (52.3)
Recruitment status		
Completed	9363 (76.5)	5758 (61.5)
Terminated	2977 (23.5)	1667 (57.9)

Abbreviations: NA, not applicable; NIH, National Institutes of Health.

^a^ The number of trials with available data might be different for different characteristics.

^b^ Including trials of devices and behavioral interventions.

### Rates of Results Reporting

**Figure 1.**
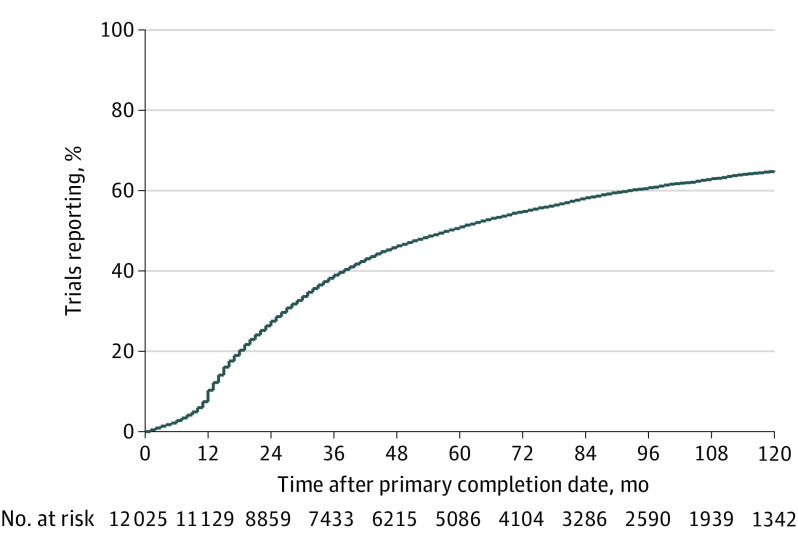
Cumulative Percentage of Clinical Trials That Reported Results on ClinicalTrials.gov or in Journal Articles

With a median (range) follow-up of 80 (41-321) months, 7425 of 12 240 trials (60.7%; 95% CI, 60.0%-61.5%) reported results. These trials enrolled a total of 760 829 participants. The results were posted on ClinicalTrials.gov for 2807 trials (22.9%; 95% CI, 22.2%-23.7%), published in journal articles for 2674 trials (21.8%; 95% CI, 21.1%-22.6%), and available both on ClinicalTrials.gov and in journal articles for 1944 trials (15.9%; 95% CI, 15.2%-16.5%). The publication date and/or the result first posted date preceded the primary completion date in 179 of 12 240 trials (1.5%; 95% CI, 1.3%-1.7%); therefore, we excluded these trials from the analyses of time to reporting but counted them as having reported results, which is consistent with a previous study.^18^ The reporting rates were 10.6% for 1 year, 27.7% for 2 years, and 51.1% for 5 years after primary completion date (Figure 1).

**Figure 2.**
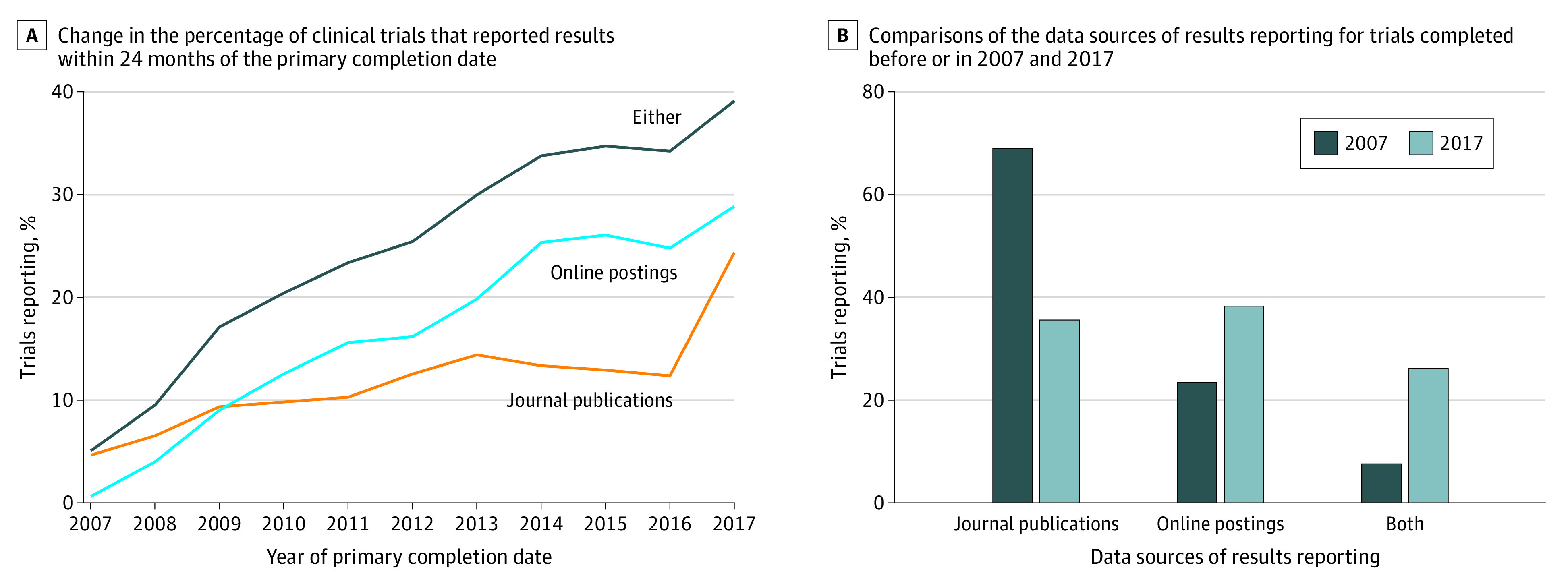
Change in the Reporting of Oncology Clinical Trials Over Time

A total of 3351 trials (27.8%; 95% CI, 26.6%-28.2%) reported results either on ClinicalTrials.gov or in journal articles within 24 months of completion. The percentage of trials that reported results within 24 months of the primary completion date increased by 34.0% (95% CI, 30.3%-37.7%) from 5.1% in or before 2007 to 39.1% in 2017. The 24-month rates of the journal publication and online posting increased over time. The rate of posting on ClinicalTrials.gov increased by 28.2% (95% CI, 25.3%-31.2%) from 0.6% in or before 2007 to 28.8% in 2017, but the rate of journal publication increased by only 19.7% (95% CI, 16.4%-23.1%) from 4.7% in or before 2007 to 24.4% in 2017. After 2009, the 24-month rate of ClinicalTrials.gov posting surpassed the rate of journal publication (Figure 2A), which led to a paradigm shift in the method of reporting. Trials completed in or before 2007 were mainly reported through journal articles (76.6%; 95% CI, 69.5%-82.7%), whereas trials completed in 2017 were mainly reported through posting on ClinicalTrials.gov (64.4%; 95% CI, 60.3%-68.4%) (Figure 2B).

### Factors Associated With Results Reporting

We used a multivariable Cox proportional hazards regression model to investigate the factors associated with results reporting (Table 2). Our findings showed that more recent trials (adjusted hazard ratio [HR], 1.11 per year increase; 95% CI, 1.10-1.13) and trials with larger sample sizes (51-100 patients: adjusted HR, 1.17 [95% CI, 1.09-1.24]; >100 patients: adjusted HR, 1.43 [95% CI, 1.33-1.54]) were more likely to report results. Terminated trials were less likely to report results compared with completed trials (adjusted HR, 0.88; 95% CI, 0.83-0.93). Compared with industry-funded trials, the NIH-funded trials were more likely to report results (adjusted HR, 1.39; 95% CI, 1.29-1.49), whereas those funded by other academic or nonprofit organizations were less likely to report results (adjusted HR, 0.66; 95% CI, 0.62-0.70).

**Table 2.** Factors Associated With Results Reporting in the Multivariable Analysis

Characteristic	Adjusted HR (95% CI)	*P* value
Primary completion year		
Per year increase	1.11 (1.10-1.13)	<.001
Registration before participant enrollment		
No	1 [Reference]	
Yes	1.31 (1.24-1.38)	<.001
Phase		
0-1	1 [Reference]	
1/2-2	2.55 (2.39-2.72)	<.001
2/3-3	2.71 (2.44-3.00)	<.001
4	1.94 (1.66-2.26)	<.001
NA^a^	1.82 (1.63-2.03)	<.001
No. of groups		
1	1 [Reference]	
2	1.18 (1.08-1.28)	.001
≥3	1.02 (0.93-1.12)	.72
Treatment randomization		
Nonrandomized	1 [Reference]	
Randomized	0.78 (0.72-0.85)	<.001
Total enrollment, No. of patients		
≤50	1 [Reference]	
51-100	1.17 (1.09-1.24)	<.001
>100	1.43 (1.33-1.54)	<.001
Funding source		
Industry	1 [Reference]	
NIH	1.39 (1.29-1.49)	<.001
Other academic or nonprofit organizations	0.66 (0.62-0.70)	<.001
Recruitment status		
Completed	1 [Reference]	
Terminated	0.88 (0.83-0.93)	<.001

Abbreviations: HR, hazard ratio; NA, not applicable; NIH, National Institutes of Health.

^a^ Including trials of devices and behavioral interventions.

### Trials That Posted Results Only on ClinicalTrials.gov

**Figure 3.**
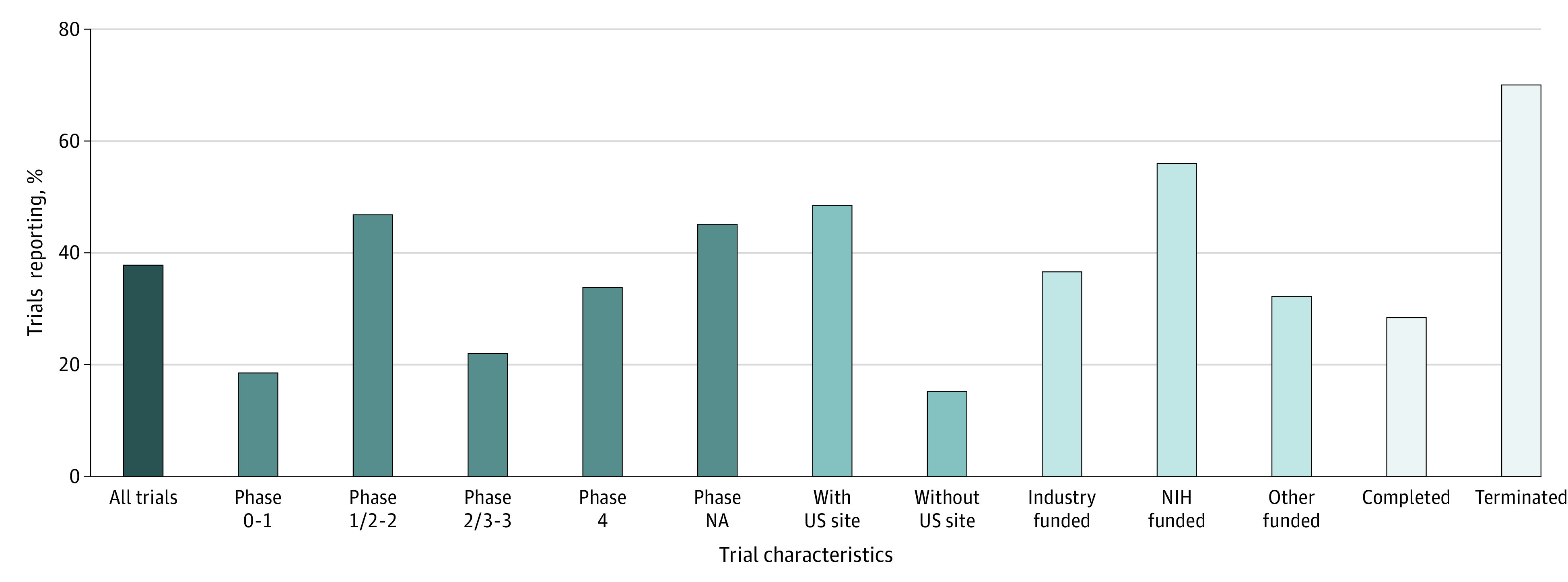
Percentage of Trials With Results Available Only on ClinicalTrials.gov NA indicates not applicable; NIH, National Institutes of Health. Other funding includes other academic or nonprofit organizations.

Among all 7425 trials that reported results, the results were posted on ClinicalTrials.gov for 2807 trials (37.8%; 95% CI, 36.7%-38.9%), published in journal articles for 2674 trials (36.0%; 95% CI, 34.9%-37.1%), and available both online and in journal articles for 1944 trials (26.2%; 95% CI, 25.2%-27.2%). The percentages of trials with results available only on ClinicalTrials.gov were different across different trial characteristics (Figure 3). For example, the results of 70.2% of the terminated trials (1171 of 1667; 95% CI, 68.0%-72.4%) vs 28.4% of the completed trials (1636 of 4122; 95% CI, 38.2%-41.2%) were available only on ClinicalTrials.gov.

Compared with trials that published results only in journal articles (n = 2674) (eFigure 2 in the Supplement), those that posted results only on ClinicalTrials.gov (n = 2807) were more likely to be terminated early (41.7% [95% CI, 39.9%-43.6%] vs 8.6% [95% CI, 7.6%-9.7%]), have a small sample size (≤50 patients: 74.6% [95% CI, 72.9%-76.2%] vs 53.2%; 95% CI, 51.3%-55.1%), have a US site (86.7% [95% CI, 85.4%- 88.0%] vs 38.8% [95% CI, 36.9%-40.7%]), be in phases 2 to 4 (82.3% [95% CI, 80.8%-83.7%] vs 58.0% [95% CI, 56.1%-59.8%]), and be funded by the NIH (19.6% [95% CI, 18.1%-21.1%] vs 5.6% [95% CI, 4.7%-6.5%]).

## Discussion

To our knowledge, this cohort study is the largest and most inclusive investigation of the reporting of oncology trial results.^9^ Despite the finding that only 60.7% of all trials reported results, we observed a promising improvement in recent years, especially for results reporting on ClinicalTrials.gov. Online trial registries could serve as a convenient platform for results reporting and an indispensable resource of data from clinical trials.

The increase in results reporting was likely associated with recent joint efforts to promote results reporting by the clinical trial enterprise, especially mandatory reporting requirements enforced by the FDAAA in the US and similar statutory regulations in Europe.^19^ Moreover, major funders, such as the NIH, require all of their sponsored trials to report results on ClinicalTrials.gov, including trials that are not covered by the FDAAA (eg, phase 1 trials).^20^ In line with this practice, we found that NIH-funded trials were more likely to report results compared with industry-funded trials (64.0% vs 55.8%; adjusted HR, 1.39), in contrast with previous reports.^9,21,22^ However, trials with funding from other academic or nonprofit organizations still lag behind (adjusted HR, 0.66).

A previous study that included 27 835 trials showed that cancer trials had the lowest rate of results reporting across all disease categories, mainly owing to the lowest rate of journal publication among these studies.^6^ A possible explanation is that oncology trials typically have small sample sizes, are not randomized or controlled, and have a high risk of yielding adverse results, which have been found to be a barrier to publishing findings in peer-reviewed journals.^4,5,6,7,23^ Therefore, posting trial results online on trial registries, independently of journal publication, is a crucial step to overcoming publication bias in oncology. For example, 70.2% of the terminated trials in this study were available only on ClinicalTrials.gov. This finding is important because terminated trials may offer potentially critical information about the lack of improved outcomes and/or adverse effects or may contradict the original assumptions of researchers and previous evidence. Publicly available results of terminated trials can prevent exposing patients to harmful or unsuccessful interventions, provide unbiased evidence of the safety and benefits of interventions, and reduce wasting limited resources.^1^ Traditionally, researchers access the results of previous studies primarily through reading journal publications. Our findings suggest that researchers contemplating new trials, systematic reviewers, and guideline developers routinely use the results database of trial registries to access all available evidence.^24,25^

Despite the improvements in results reporting in recent years, additional strategies to increase trial transparency are needed for several reasons. First, the US National Library of Medicine, which operates and maintains ClinicalTrials.gov, cannot verify the validity of all submitted results. Although it is likely not feasible to apply the peer-review process to submitted results, it is possible for ClinicalTrials.gov to allow readers to comment on the results and ask the investigators to respond. This practice might improve the completeness and quality of the results reported on ClinicalTrials.gov. Second, the academic community needs to acknowledge the contributions of researchers who post trial results on ClinicalTrials.gov (eg, listing the names of investigators and citing their results). Third, the World Health Organization should encourage all registries on its International Clinical Trials Registry Platform to promote results reporting and establish minimum requirements for this process; similar requirements have been developed for trial registration.^26^ These steps will promote standardized reporting of trial results across different registries worldwide; standardization will become even more important as trials in emerging markets rapidly increase.^6^

### Limitations

This study has some limitations. First, we did not inspect whether trials were registered and reported results on other trial registries. Second, this analysis relied on information provided to ClinicalTrials.gov by the responsible parties; thus, missing and/or inaccurate data may have affected the conclusions of this study. For example, the publication date and/or result first posted date preceded the primary completion date in 1.5% of the trials, which was likely attributed to the existence of multiple primary end points or input errors or to a misunderstanding of the definitions.^7,18^ Third, similar to previous studies, our method of linking registered trials to associated publications relied on the presence of an NCT number in the text or metadata of the journal article,^6,15^ which may have led to our missing some reported trials. However, medical journals encourage the reporting of registration numbers in articles through the policy of the International Committee of Medical Journal Editors and the Consolidated Standards of Reporting Trials, and it can be argued that compliance is part of the investigators’ responsibility to ensure that their research is transparent and discoverable.^27,28^ Moreover, PubMed has been indexing publications using NCT numbers since July 2005. Thus, although the absolute reporting rates might have been affected, the comparative analysis presented in this study is unlikely to have been biased toward either of the categories (eg, the funding source or recruitment status of completed vs terminated).

## Conclusions

This cohort study found a promising increase in the reporting of oncology trial results over a 10-year period; however, it also found room for future improvement. Trial registries could serve as a convenient platform for results reporting and as an indispensable resource of data from clinical studies.

## Notes

Supplement. **eFigure 1.** Flowchart Identifying Trials Registered on Clinicaltrials.gov in 2007-2017 and Completed Before October 1, 2017 **eFigure 2.** Characteristics of Trials That Reported Results Only in Journal Publications (n = 2674) Versus Those That Reported Results Only on ClinicalTrials.gov (n = 2807)
