# Supplementary material for: Evaluation of Oncology Trial Results Reporting Over a 10-Year Period
Source: JAMA Netw Open. 2021 May 24;4(5):e2110438. doi: 10.1001/jamanetworkopen.2021.10438 (PMC8144925; doi:10.1001/jamanetworkopen.2021.10438)
Supplement: Supplement. — eFigure 1. Flowchart Identifying Trials Registered on Clinicaltrials.gov in 2007-2017 and Completed Before October 1, 2017 eFigure 2. Characteristics of Trials That Reported Results Only in Journal Publications (n = 2674) Versus Those That Reported Results Only on ClinicalTrials.gov (n = 2807) [file jamanetwopen-e2110438-s001.pdf]

Supplementary Online Content

Liu X, Zhang Y, Li WF, et al. Evaluation of oncology trial results reporting over a 10-year period. *JAMA Netw Open*. 2021;4(5):e2110438. doi:10.1001/jamanetworkopen.2021.10438

eFigure 1. Flowchart Identifying Trials Registered on Clinicaltrials.gov in 2007-2017 and Completed Before October 1, 2017

eFigure 2. Characteristics of Trials That Reported Results Only in Journal Publications (n = 2674) Versus Those That Reported Results Only on ClinicalTrials.gov (n = 2807)

This supplementary material has been provided by the authors to give readers additional information about their work.

© 2021 Liu X et al. *JAMA Network Open.*

eFigure 1. Flowchart Identifying Trials Registered on Clinicaltrials.gov in 2007-2017 and Completed Before October 1, 2017

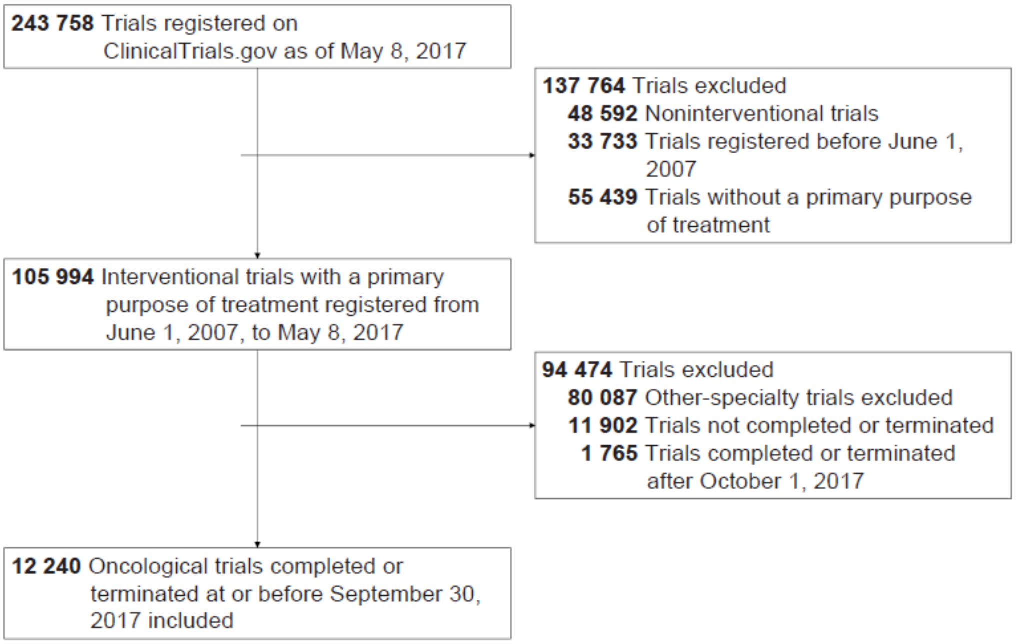

© 2021 Liu X et al. *JAMA Network Open.*

eFigure 2. Characteristics of Trials That Reported Results Only in Journal Publications (n = 2674) Versus Those That Reported Results Only on ClinicalTrials.gov (n = 2807)

Other sponsorships denote trials sponsored by other academic or non-profit organizations. Prospectively registered denotes trials registered before enrolling the first participant. NIH, National Institutes of Health; US, United States

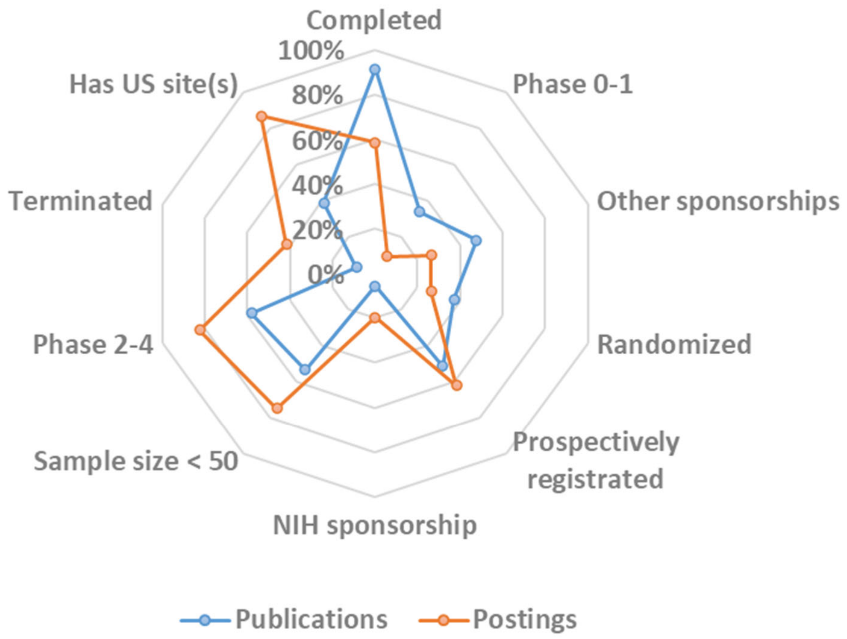

© 2021 Liu X et al. *JAMA Network Open.*
